# Repair of postoperative defects of oral cancer with submental island flaps based on vascular pedicles of different states: a multicenter retrospective study

**DOI:** 10.1038/s41598-022-24293-4

**Published:** 2022-12-08

**Authors:** Jiuzhou Zhao, Wen Zeng, Ke Li, Jing Huang, Michael C. F. Tong, Lifei Deng, Xiangmin Zhang

**Affiliations:** 1 Department of Otolaryngology, Longgang E.N.T Hospital, Shenzhen Key Laboratory of E.N.T, Institute of E.N.T Shenzhen, No. 3004, Longgang Avenue, Shenzhen, Guangdong People’s Republic of China; 2 Department of Head and Neck, Tumor Hospital of Ganzhou, Ganzhou, Jiangxi People’s Republic of China; 3 Department of Institute of Cancer Research, Tumor Hospital of Ganzhou, Ganzhou, Jiangxi People’s Republic of China; 4 Department of Otorhinolaryngology, Head and Neck Surgery, The Chinese University of Hong Kong, Shatin, New Territories, Hong Kong, SAR People’s Republic of China; 5 Department of Head and Neck, Jiang Xi Caner Hospital, No. 519, Beijing East Road, Nanchang, Jiangxi People’s Republic of China

**Keywords:** Cancer, Head and neck cancer, Oral cancer

## Abstract

Submental island flap has certain advantages in repairing postoperative defects of oral cancer, and it can often achieve similar or even better effects compared with those of the free tissue flap. In this study, according to the different characteristics of patients and postoperative defects of oral cancer, submental island flaps with different states of vascular pedicle were prepared, and its repair methods, safety, and clinical effects in treating postoperative defects of oral cancer were investigated. 83 patients with oral cancer who met the inclusion criteria were selected. According to the different characteristics of the patients and postoperative defects of oral cancer, the traditional submental island flap vascular pedicle was modified into three different states: submental artery perforator flap, vascular pedicled flap with the anterior belly of digastric muscle but without the submandibular gland (SIF with anterior belly of DM), and vascular pedicled flap with the anterior belly of the digastric muscle and the submandibular gland (SIF with anterior belly of DM and SG). The types of the submental artery and the drainage vein, flap survival, and complications, were observed. The flap was successfully harvested for all patients, and the submental artery could be found or separated for all of them, with the venous drainage to the internal jugular vein in 57 (57/83, 68.67%), to the external jugular vein in 18 (18/83, 21.69%), and to the anterior jugular vein in eight (8/83, 9.64%) cases. Submental artery perforator flap was used for 11 cases, complete necrosis occurred in two cases (2/11, 18.18%), partial necrosis occurred in one case (1/11, 9.09%); SIF with anterior belly of DM was used for 49 cases, complete necrosis occurred in one case (1/49, 2.04%), partial necrosis occurred in four cases (4/49, 8.16%); SIF with anterior belly of DM and SG was used for 23 cases, including chimeric flap combining the submental island flap and the submandibular gland used for 15 cases, there were no cases of complete or partial necrosis. Submental island flap was effective in repairing postoperative defects of oral cancer. Submental island flaps with three different states of vascular pedicle could repair oral cancer-affected tissues with different defect characteristics.

## Introduction

The incidence of head and neck tumors has been increasing year by year, especially the incidence of oral cancer has been high, and surgery is the main means to treat oral cancer^1–3^. Postoperative defects of oral cancer cause internal and external deformities of patients, which seriously affect their speech, swallowing and other functions^4,5^. Therefore, it is difficult for head and neck oncologists to completely remove oral tumors, immediately repair defects, restore the appearance and function of patients, reduce the physiological, psychological, and social impact on patients with oral cancer, and improve the quality of life^6,7^. Free flaps, such as anterior arm flap, lateral upper arm flap and anterolateral thigh flap, could achieve satisfactory appearance reconstruction effect, but microvascular anastomosis technology, as well as postoperative flap observation and nursing, have limited the wide applicability of free flaps^8–10^. In recent years, the submental island flap (SIF), supra clavicular flap, and other pedicled flaps have shown similar or even better repair effects than free flaps. SIFs were clinically safe and reliable, and had more advantages in terms of the operation time, the average length of hospital stay, cost, etc.^11,12^.

The SIF is adjacent to the defect area of the face and neck, needs simple preparation, has a rich blood supply, and has similar texture and color to the defect area; therefore, it has been widely used in the field of repair and reconstruction^13,14^. During the preparation of the SIF, to protect the submental artery and vein, surgeons are often afraid, unwilling, or unable to adequately clean the lymphatic and adipose tissues at level I, which limits the development of SIFs in head and neck tumor surgery, especially in oral cancer^15,16^. With the rise of pedicled perforator flaps and the wide application of chimeric flaps, according to the patients’ conditions and different characteristics of postoperative defects of oral cancer, we modified the traditional SIF vascular pedicle into three different states: submental artery perforator flap, vascular pedicled flap with the anterior belly of digastric muscle but without the submandibular gland (SIF with anterior belly of DM), and vascular pedicled flap with the anterior belly of the digastric muscle and the submandibular gland (SIF with anterior belly of DM and SG). We observed the types of the submental artery and drainage vein, flap survival and complications, and quality of life, and further summarized the clinical experience to enrich and develop SIFs.

## Materials and methods

### Ethics statement

The Ethical Committee of the Longgang E.N.T hospital approved clinical samples for research purposes (No. 2022-0002), and this study was confirmed to the principles contained in the World Medical Association Declaration of Helsinki. Informed consent was obtained from all subjects.

### Case inclusion and exclusion criteria

Inclusion criteria: ① a clear pathological diagnosis of oral cancer; ② age ≥ 35 years; ③ All patients were treated for the first time; ④ tumor T stage < T4; ⑤ no serious cardiovascular or cerebrovascular diseases, diabetes, or chronic respiratory diseases; ⑥ Karnofsky score ≥ 80 points; ⑦ Estimated survival more than 1 year; ⑧ patients who voluntarily signed the informed consent form.

Exclusion criteria: ① lymph node metastasis at bilateral level I or bilateral cervical examined by imaging before operation; ② lymph node metastasis found by intraoperative lymph node dissection at Level I and frozen pathology; ③ lesions found in the submental regions; ④ history of neck surgery or radiotherapy; ⑤ distant metastasis; ⑥ difficulty in tolerating surgery due to surgical contraindications such as poor general condition, or cardiovascular or cerebrovascular diseases.

### Selection of vascular pedicle

To repair the distant medium and small superficial defects, a perforator flap was chosen. For patients with deep invasion by the primary lesion or large defects, or those at an advanced age or in poor health, SIF with anterior belly of DM or SIF with anterior belly of DM and SG can be chosen for repair. The anterior belly of the digastric muscle and the submandibular gland can be used to repair the mouth floor or other closed defects. If necessary, the submandibular gland and the SIF can be formed into a chimeric flap to repair larger oral defects.

### Clinical data

This study is a multicenter retrospective study. From July 2011 to June 2021, 83 patients with oral cancer who met the inclusion criteria were selected from the Department of Head and Neck of Shenzhen Institute of Otolaryngology/Shenzhen Longgang Otolaryngology Hospital, the Department of Head and Neck of the affiliated cancer hospital of Gannan Medical University, and the Department of Head and Neck of the affiliated cancer hospital of Nanchang University. There were 60 males and 23 females. Their age range was 35–69 years, the average age is 55.7 years. Among them, there were 37 cases of tongue cancer, 21 of gingival cancer, 13 of buccal cancer, and 12 of mouth floor cancer. There were 23 cases at Stage II, 34 at Stage III, 19 at Stage IVA, and seven at Stage IVB (Table 1).

**Table 1:** General clinical data of the patients (n = 83).

Parameter	Number of patients, n (%)
**Age, years**	
30–39	3 (3.62)
40–49	14 (16.87)
50–59	37 (44.58)
60–69	29 (34.93)
**Sex**	
Male	60 (72.29)
Female	23 (27.71)
KPS	
80–89	14 (16.87)
≥ 90	69 (83.13)
**Type of tumor**	
Tongue cancer	37 (44.58)
Gingival cancer	21 (25.30)
Buccal cancer	13 (15.66)
Mouth floor cancer	12 (14.46)
**TNM stages**	
II	23 (27.72)
III	34 (40.96)
IVA	19 (22.89)
IVB	7 (8.43)

*KPS* karnofsky performance score.

### Surgical procedure

The surgery included selective or total cervical lymph node dissection, extended lesion resection, SIF preparation, defect repair, reconstruction of appearance and function, and other steps.

**Figure 1:**
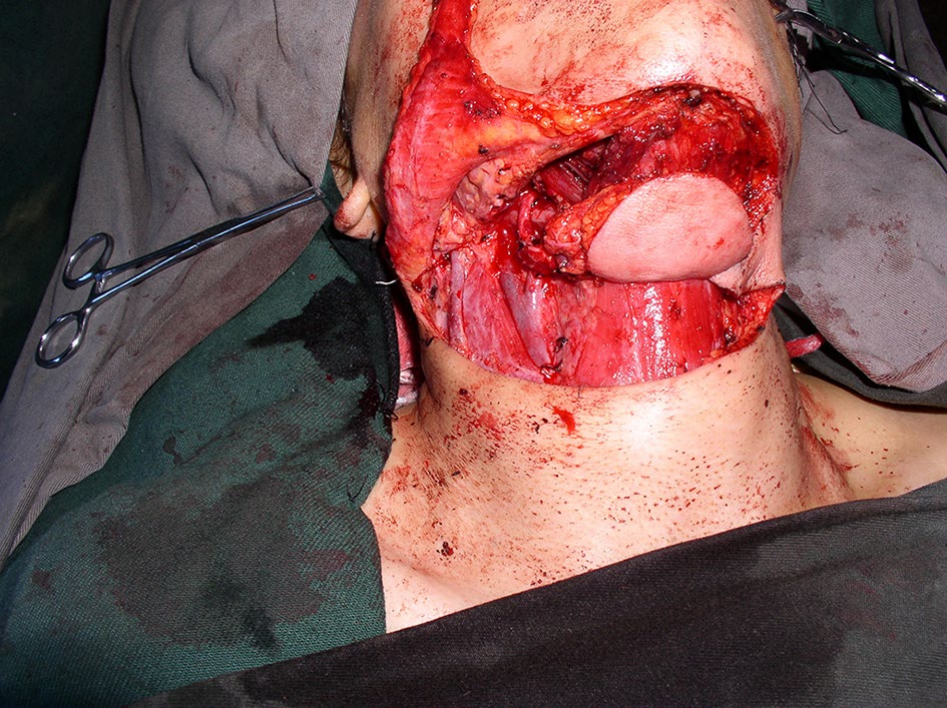
Submental artery perforator flap without the anterior belly of the digastric muscle and the submandibular gland for the vascular pedicle.

**Figure 2:**
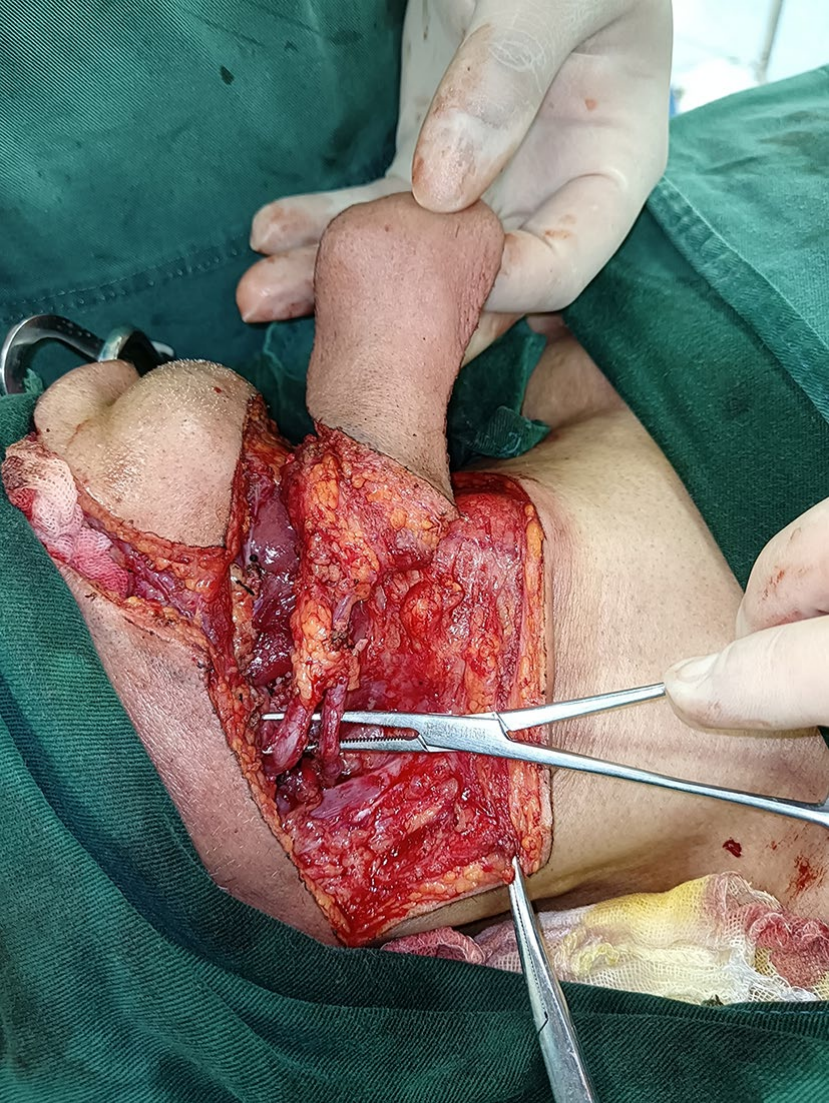
Submental island flap with the anterior belly of the digastric muscle but without the submandibular gland for the vascular pedicle.

**Figure 3:**
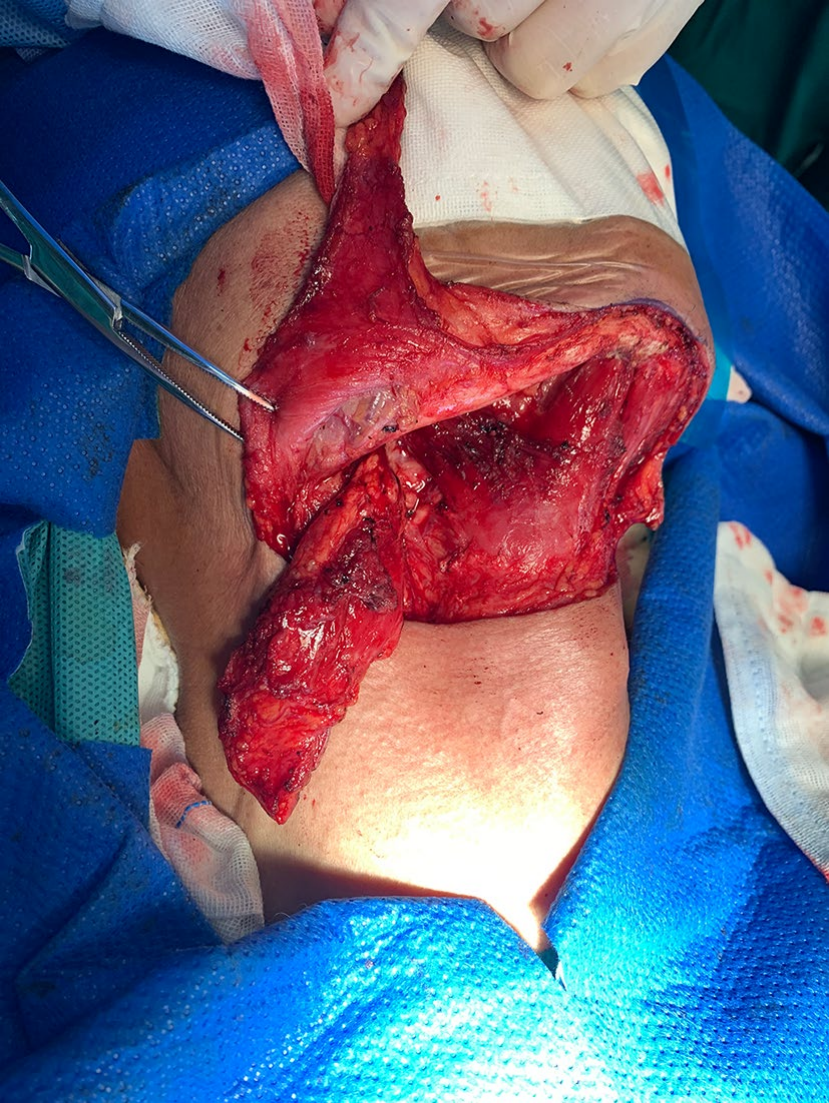
Submental island flap with the anterior belly of the digastric muscle and the submandibular gland for the vascular pedicle.

The size of the cutaneous flap was designed according to the estimated defect area after enlarged resection, and the skin, subcutaneous tissue and platysma muscle were cut open according to the designed incision. The mandibular marginal branch of the facial nerve was searched and protected between the deep surface of the platysma muscle and the superficial layer of the deep cervical tendon, in the inferior edge of the mandible, and in the mandibular angle. The distal ends of the facial artery and vein were ligated at the intersections of the mandibular marginal branch of the facial nerve and distal ends of the facial artery and vein to avoid damaging the submental artery. According to each patient’s condition and the different characteristics of postoperative defects of oral cancer, SIFs with different states of the vascular pedicle was selected. For the perforator flap, the vascular pedicle was exposed without muscles and the submandibular gland while the arteries and veins were carefully protected (Fig. 1). For the SIF with anterior belly of DM, the terminal branches of submental blood vessels on the deep surface of the anterior belly of the digastric muscle should be protected (Fig. 2). For SIF with anterior belly of DM and SG, the branching vessels of the submandibular glands should be carefully protected and provided nutrition (Fig. 3). For SIF with anterior belly of DM and SG, the submandibular gland should be used to form a chimeric flap or to close the tissue defect in the operative cavity according to the size of the repaired area and the amount of tissue defect in the operative cavity^17^.

**Figure 4:**
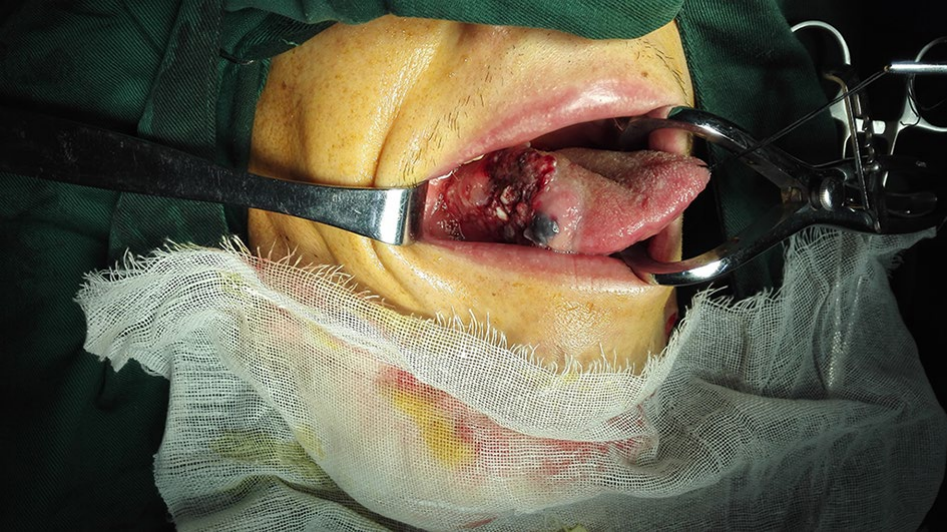
Squamous cell carcinoma on the right side of the tongue.

**Figure 5:**
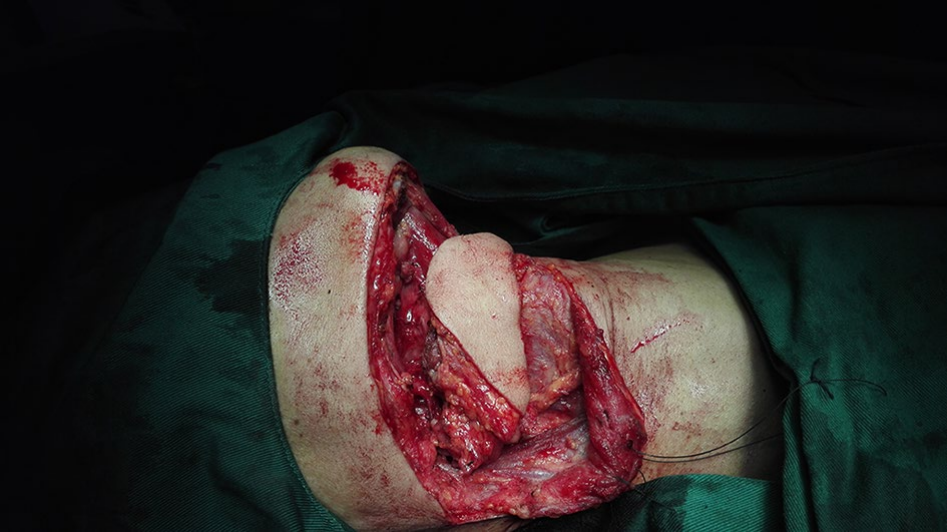
Submental island flap prepared at the same time of cervical lymphadenectomy.

**Figure 6:**
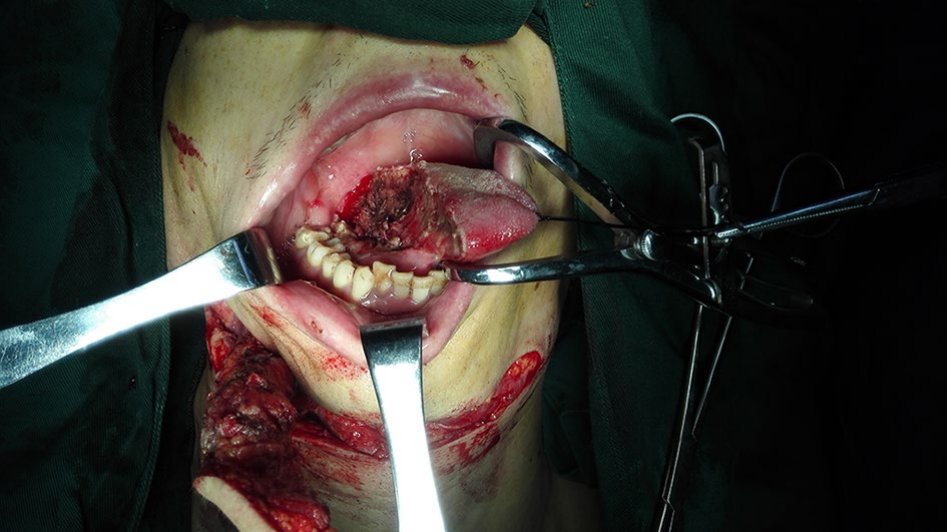
Half tongue defect after primary lesion resection of tongue squamous cell carcinoma.

**Figure 7:**
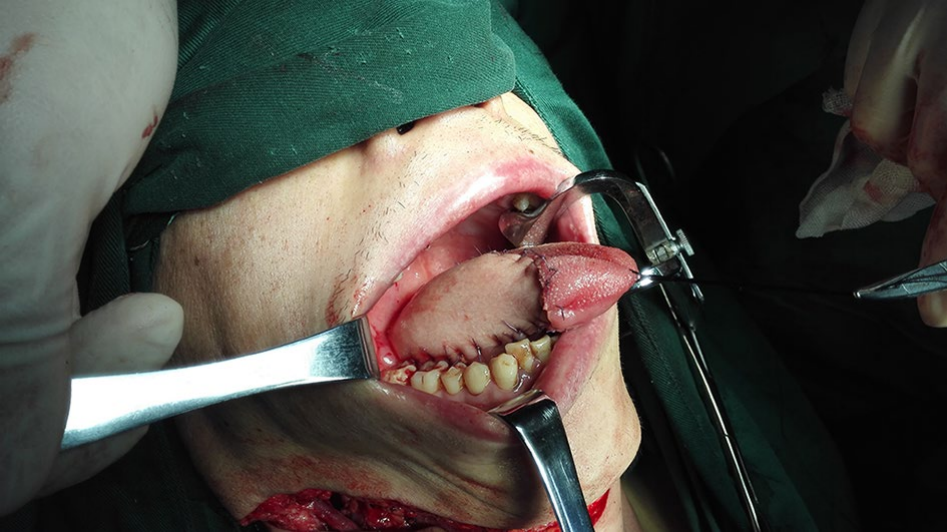
Postoperative state of hemi-tongue defect repaired by submental island flap.

**Figure 8:**
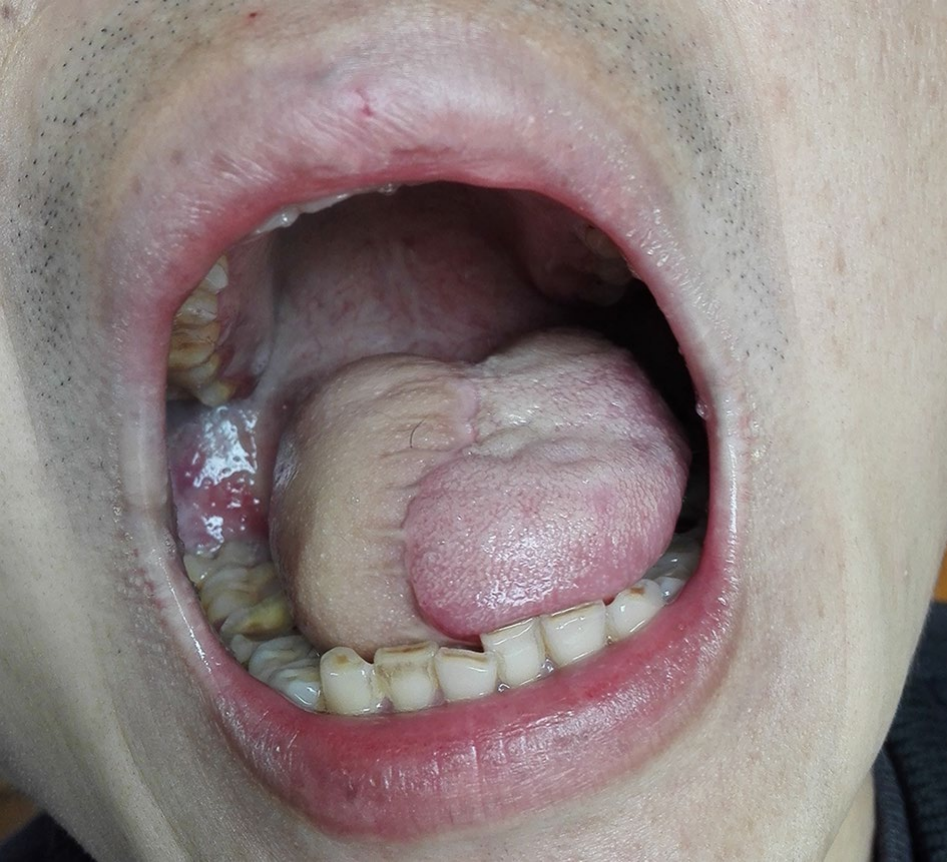
Six months after the hemi-tongue defect repair by submental island flap, with the full appearance of the tongue.

For all types of flaps, concurrent with the preparation, lymphatic and adipose tissues should be carefully cleaned according to standard protocols, lymphatic and adipose tissues should be sent for fast freezing, absence of lymph node metastasis should be confirmed, and the flap should be transferred to the oral cavity to repair the defect (Figs. 4, 5, 6, 7, and 8).

### Postoperative radiotherapy

Postoperative adjuvant radiotherapy for Stage T3 cancer was performed in the lymphatic drainage area of the neck according to the status of lymph nodes. Routine facial-cervical combined field + cervical supraclavicular tangent field irradiation was performed. The upper bound was located 1.5–2 cm above the lingual surface when the mouthpiece was placed in the patient’s mouth, the lower bound was at the level of the lower edge of the hyoid bone, the anterior bound included the anterior edge of the genion avoiding the lower lip, and the posterior bound was at the posterior edge of the spinous process; the postoperative radiotherapy dose was dose of total (DT) 60GY/30F/6W. Intensity-modulated radiation therapy, target volume, gross tumor volume bed (GTV-bed), the tumor scope indicated by preoperative imaging and clinical examination, and the operative cavity after surgery; clinical target volume (CTV): the GTV-bed and its surrounding adjacent soft tissues and the lymphatic drainage area of the neck; dose: GTV-bed 59.36 Gy/28F; CTV 50.4/28F, if the surgical margin was positive or close to the tumor, the radical radiotherapy dose was adopted, GTV66-70GY/31-33F.

### Observation items

During the operation, the venous drainage of the flap was observed. After the operation, flap survival, wound healing, complications, surgical margin, and pathological status of cervical lymph nodes were noted. After repair, the appearance and function of the oral cavity, as well as radiotherapy tolerance were observed. Moreover, the recurrence of the primary lesion and cervical lymph nodes were noted.

### Statistical methods

The complete necrosis, partial necrosis, postoperative recurrence, and other conditions of SIFs with three different states of the vascular pedicle were statistically analyzed. The Chi-square test was performed using IBM SPSS 22.0, and the difference was considered statistically significant for *P* < 0.05.

### Ethics approval and consent to participate

This retrospective chart review study involving human participants was in accordance with the ethical standards of the institutional and national research committee and with the 1964 Helsinki Declaration and its later amendments or comparable ethical standards. The Ethical Committee of the Longgang E.N.T hospital & Shenzhen Key Laboratory of E.N.T & Institute of E.N.T Shenzhen Review Board approved the study protocol (2022-0002) and written informed consent was harvested from participants.

## Results

### Arterial travel and venous drainage of the flap

Among the 83 cases using SIFs, submental artery perforator flap was used for 11 cases; SIF with anterior belly of DM was used for 49 cases; SIF with anterior belly of DM and SG was used for 23 cases, including chimeric flap combining the SIF and submandibular gland used for 15 cases. The flap was successfully harvested for all patients, and the submental artery could be localized or separated for all of them, the submental artery starts from the facial artery on the deep surface of the submandibular gland and runs forward. The medial side is the mylohyoid muscle, the upper side is the lower margin of the mandible, and reaches the anterior belly of the digastric muscle, there are small branches to the submandibular gland, mylohyoid muscle, digastric muscle, mandibular periosteum and other places along the way. The terminal branch fuses with the contralateral submental artery in the submental region. There was a lot of variation in venous drainage, with a venous drainage to the internal jugular vein in 57 cases (57/83, 68.67%), to the external jugular vein in 18 (18/83, 21.69%), and the anterior jugular vein in eight (8/83, 9.64%) (Table 2).

**Table 2:** Venous reflux of submental island flap (n = 83).

Location	Number of patients, n (%)
Internal jugular vein	57 (66.87)
External jugular vein	18 (21.69)
Anterior jugular vein	8 (9.64)

### Survival of flap

Among the 83 cases using SIFs, complete survival of the flap occurred in 75 (75/83, 90.36%), complete necrosis occurred in three (3/83, 3.62%), and partial necrosis occurred in five (5/83, 6.02%) cases. Complete necrosis of submental artery perforator flap occurred in two cases (2/11, 18.18%), partial necrosis of the submental artery perforator flap occurred in one case (1/11, 9.09%); complete necrosis of SIF with anterior belly of DM occurred in one case (1/49, 2.04%), partial necrosis of SIF with anterior belly of DM occurred in four cases (4/49, 8.16%); there was no complete necrosis and no partial necrosis of SIF with anterior belly of DM and SG (0/23, 0.00%), with statistically significant differences (*P* < 0.05) (Table 3).

**Table 3:** Survival of skin flap (n = 83).

Parameter	Number of patients, n (%)	Complete survival, n (%)	Necrosis, n (%)	
			Complete necrosis, n (%)	Partial necrosis, n (%)
Submental artery perforator flap	11 (13.25)	8 (72.73)	2 (18.18)	1 (9.09)
SIF with anterior belly of DM	49 (59.04)	44 (89.80)	1 (2.04)	4 (8.16)
SIF with anterior belly of DM and SG	23 (27.71)	23 (100.00)	0 (0.00)	0 (0.00)
*P*	0.0408			
χ^2^	6.3988			

### Complications, surgical margin, and pathological state of lymph nodes at Level I

5 patients with partial necrosis of the flap were cured after dressing change, and 3 patients with complete necrosis of the flap were operated again, the necrotic flap was removed and sutured directly. There were no surgery-related deaths and no operative complications, and primary healing was achieved in all patients’ incisions. The surgical margin was negative in 83 patients. A total of 209 lymph nodes were removed from Level I during the operation. There was no cancer metastasis found by intraoperative fast freezing or postoperative paraffin section examination.

### Follow-up status

Follow-up was conducted from the end of treatment to December 31, 2021, and all 83 patients were followed up for 6–123 months. The local appearance and function recovered well. No patient had significant discomfort in the submental region. The flap of patients undergoing radiotherapy could tolerate during and after radiotherapy, and there was no discontinuation of radiotherapy due to flap necrosis. There was a total of seven cases of local recurrence; no recurrence of perforator flap (0/11, 0.00%), recurrence of SIF with anterior belly of DM in three cases (3/49, 6.12%) and recurrence of SIF with anterior belly of DM and SG in four cases (4/23, 17.39%), without statistically significant differences (*P* > 0.05; χ^2^ = 3.7419, *P* = 0.1540) (Table 4).

**Table 4:** Tumor recurrence (n = 83).

Parameter	Number of patients, n (%)	Tumor recurrence, n (%)
Submental artery perforator flap	11 (13.25)	0 (0.00)
SIF with anterior belly of DM	49 (59.04)	3 (6.12)
SIF with anterior belly of DM and SG	23 (27.71)	4 (17.39)
*P*		0.1540
χ^2^		3.7419

## Discussion

The incidence of oral cancer has been high, and comprehensive surgical treatment remains the main treatment approach. Tissue flap repair and reconstruction technology play an important role in extended tumor resection, postoperative reconstruction, restoration of oral function, alleviation of physiological, psychological and social impact, and improving quality of life. With the development of head and neck functional surgery, a consensus has been attained on the immediate postoperative repair and reconstruction of oral cancer that free flap can achieve satisfactory appearance reconstruction and functional recovery, and is the primary means for repairing postoperative defects of oral cancer^8–10,18^. The high-level technique required for preparation of free flap, the delicate and complicated operation of microvascular anastomosis, the long operation time, the high risk if a vascular crisis occurs, and the perioperative nursing have limited the wide application of free flap^19,20^. SIFs have the advantages of moderate thickness, similar color to the defect site, small surgical trauma, short operation time, reliable blood supply, easy survival, no need of vascular anastomosis, and small defect of the donor site, making it deeply favored by oral and maxillofacial surgeons as well as head and neck oncologists^21,22^.

The SIF takes the submental artery as its blood supply artery, and the pedicle can be as long as 8 cm. SIF can be made into an axial or free flap, which can be rotated at a large angle, and can be used as a flap, myofascial flap, or musculoskeletal flap^23,24^. The location of the submental artery is relatively constant, and there are branches along the way to the submandibular gland, platysma muscle, digastric muscle, mylohyoid muscle, and subcutaneous region, with many variations in submental venous drainage, which can easily lead to congestion and necrosis of the flap due to accidental injury during operation^25,26^. This study identified the venous drainage to the internal jugular vein in 68.67% of the cases, to the external jugular vein in 21.69% of the cases, and the anterior jugular vein in 9.64% of the cases. In order to ensure venous drainage, the lymphatic and adipose tissues at Level I should be resected during the operation, and the venous network in this region should be preserved as much as possible. According to each patient’s condition and the different characteristics of postoperative defects of oral cancer, we modified the SIF vascular pedicle into three different states: submental artery perforator flap, SIF with anterior belly of DM, and SIF with anterior belly of DM and SG.

With the thriving of a pedicled perforator flap, the SIF can be taken as a perforator flap, which does not carry the anterior belly of the digastric muscle and the submandibular gland, and the vascular pedicle is thinner and longer, which can repair the distant medium and small superficial defects. At the same time, the local recurrence rate is also low. The disadvantage is that as it takes a long operation time to harvest the flap, the perforator vessel can be easily damaged. Hence it is not easy for beginners to master, and it is prone to venous drainage disorder after the operation. To better protect the perforator vessel and reduce the risk of venous drainage disorder, the anterior belly of the digastric muscle can be retained on the vascular pedicle after harvesting the SIF, which can be used for repair in patients with deep invasion and close distance of the primary lesion. For patients with deep invasion of the primary lesion and large defects, the anterior belly of the digastric muscle and the submandibular gland can be preserved when harvesting the SIF, which can reduce the time for harvesting the flap, vascular pedicle injury and venous drainage disorder. Concurrently, the anterior belly of the digastric muscle and the submandibular gland can be used to repair the mouth floor or other closed defects, and if necessary, the submandibular gland and SIF can be formed into a chimeric flap to repair larger oral defects^17^. In this study, the anterior belly of the digastric muscle and the submandibular gland were preserved when harvesting SIFs in 23 patients, the submandibular gland was used to repair closed defects in eight cases, and the submandibular gland and SIF were combined to form a chimeric flap in 15 cases.

Whether the submandibular gland can be preserved for the vascular pedicle of the SIF depends on the tumor metastasis or lymph node metastasis in the submandibular gland. The presence of lymph nodes in the submandibular gland is still controversial. DiNardo^27^ believed that there are no lymph nodes in the submandibular gland. A large number of studies have confirmed that it is safe and feasible to preserve the submandibular gland in total cervical lymphadenectomy administered for oral and oropharyngeal cancer. The involvement of the submandibular gland is mainly by the direct invasion of the tumor or invasion by lymph nodes around the gland^28,29^. Therefore, in the vascular pedicle of the SIF, it is safe and feasible to preserve the submandibular gland after total dissection of lymphatic and adipose tissues at Level I^30,31^. In our multicenter retrospective study, no flap necrosis occurred in SIFs with the submandibular glands preserved for the vascular pedicle, which exhibited superior advantages compared to those associated with the perforator flap and SIF without preserving the submandibular gland for the vascular pedicle. The flap preparation time was further shortened, and the safety was greatly improved. Moreover, the submandibular gland could repair closed defects as needed, or it could be combined with the SIF to repair a wider scope of defects, and there was no significant difference in the recurrence rate.

In the 83 cases using SIFs, the complete survival rate was 90.36%, complete necrosis rate was 3.62%, partial necrosis rate was 6.02%, and necrosis rate was 9.64%; the recurrence rate was 8.43%. The relatively low flap necrosis rate and recurrence rate verified our correct choice. After a thorough preoperative evaluation, the detailed operation plan and alternative flap repair plan were established. According to the preoperative planning and the operator’s proficiency, the appropriate vascular pedicle was selected. Regardless of the kind of vascular pedicle selected, it is necessary to avoid damaging the submental artery and vein when harvesting the flap. The SIF artery has a constant location, while variations of the vein are large, which is prone to venous crisis. In this study, three cases of complete necrosis were associated with venous drainage disorder, and five of partial necrosis were also associated with veins. To harvest SIFs, the lymphatic and adipose tissues at Level I should be completely resected. During the resection, the electrotome should keep a safe distance from the vascular pedicle. Separation and removal of the submandibular gland should be gentle, so to protect the drainage vein. Concurrently, the lymphoid adipose tissue at Level I should be sent for frozen pathology. If the lymph nodes are positive, the ipsilateral SIF cannot be used, and other flaps can be used for repair, or SIFs with contralateral vascular pedicles can be used.

Patients with oral cancer require comprehensive treatment, and individualized comprehensive treatment plans should be made under multi-disciplinary combined diagnosis and treatment. According to the principles of head and neck functional surgery, on the premise of ensuring the resection of diseased tissues, the normal structures should be preserved to the maximum extent and the defect area should be repaired and reconstructed, to achieve the purpose of repairing function and esthetics. A rigorous surgical plan should be tailored, and the most suitable surgical plan and reconstruction approach should be selected after the doctor and patient reach an agreement according to the patient’s condition and needs. When choosing SIF repair and reconstruction, SIFs with different vascular pedicles should be selected according to each patient’s condition and the different characteristics of postoperative defects of oral cancer. Strict observation and evaluation of the treatment outcome should be conducted after the operation. Once recurrence is identified, multidisciplinary combined diagnosis and treatment should be provided again as soon as possible to determine the most favorable treatment plan.

## Conclusion

Submental island flap was effective in repairing postoperative defects of oral cancer. Submental island flaps with three different states of vascular pedicle could repair oral cancer-affected tissues with different defect characteristics. Perforator flap without the anterior belly of the digastric muscle and the submandibular gland had a thinner and longer vascular pedicle; it could repair medium and small superficial defects farther away and had a low local recurrence rate. However, the disadvantage of this flap was that venous drainage disorder could easily occur. For patients with deep invasion by the primary lesion or large defects, vascular pedicled flap with the anterior belly of digastric muscle but without the submandibular gland or vascular pedicled flap with the anterior belly of the digastric muscle and the submandibular gland can be chosen for repair. The anterior belly of the digastric muscle and the submandibular gland can be used to repair the floor of the mouth or other closed defects. If necessary, the submandibular gland and the submental island flap can be formed into a chimeric flap to repair larger oral defects, with a high survival rate of the flap, but there is a risk of local recurrence.

## Data Availability

Datasets are available on request from the corresponding author on reasonable request. The raw data and all related documents supporting the conclusions of this manuscript will be made available by the authors,without undue reservation, to any qualified researcher.
